# Impact of beliefs on perception of newborn illness, caregiver behaviors, and care-seeking practices in Zambia’s Southern province

**DOI:** 10.1371/journal.pone.0282881

**Published:** 2023-05-25

**Authors:** Kasthuri Sivalogan, Bowen Banda, John Wagner, Godfrey Biemba, Natalie Gagne, Caroline Grogan, Fern Hamomba, Julie M. Herlihy, Catherine Mabeta, Peggy Shankoti, Grace Simamvwa, Bernadine Sooli, Kojo Yeboah-Antwi, Davidson H. Hamer, Katherine E. A. Semrau

**Affiliations:** 1 Emory Global Health Institute at Emory University, Atlanta, Georgia, United States of America; 2 Department of Global Health, Boston University School of Public Health, Boston, Massachusetts, United States of America; 3 Zambian Center for Applied Health Research and Development, Limited, Lusaka, Zambia; 4 Department of Internal Medicine, Rush University Medical Center, Chicago, Illinois, United States of America; 5 National Health Research Authority, University Teaching Hospital Paediatric Centre of Excellence, Lusaka, Zambia; 6 Canadian Federal Department of Indigenous Services Canada, Gatineau, Canada; 7 Ariadne Labs, Harvard T.H Chan School of Public Health, Brigham and Women’s Hospital, Boston, Massachusetts, United States of America; 8 Department of Pediatrics, Boston University School of Medicine, Boston, Massachusetts, United States of America; 9 Department of Medicine, Section of Infectious Diseases, Boston Medical Center, Boston, Massachusetts, United States of America; 10 Department of Medicine, Harvard Medical School, Boston, Massachusetts, United States of America; 11 Division of Global Health Equity, Brigham and Women’s Hospital, Boston, Massachusetts, United States of America; Medical Research Council, SOUTH AFRICA

## Abstract

Despite reductions in the number of under-five deaths since the release of the Sustainable Development Goals, the proportion of neonatal deaths among all under-five deaths has remained high. Neonatal health is linked to newborn care practices which are tied to distinct cultural perceptions of health and illness. We assessed how community beliefs in Zambia’s Southern Province influence newborn care behaviors, perception of illness, and care-seeking practices, using qualitative data collected between February and April 2010. A total of 339 women participated in 36 focus group discussions (FGDs), with 9 FGDs conducted in each of the four study districts. In addition, 42 in-depth interviews (IDIs) were conducted with various key informants, with 11 IDIs conducted in Choma, 11 IDIs in Monze, 10 IDIs in Livingstone, and 10 IDIs in Mazabuka. The FGDs and IDIs indicate that beliefs among the Tonga people regarding postnatal illness prevention and management influence perceptions of newborn illness and care-seeking practices. Care seeking behaviors including when, why, and where parents seek newborn care are intimately tied to perception of disease among the Tonga people. These beliefs may stem from both indigenous and Western perspectives in Zambia’s Southern Province. Findings are consistent with other analyses from Southern Province that highlighted the benefit of integrating local practices with Western biomedical care. Health systems models, led by policy makers and program designers, could aim to find synergies between community practices and formal health systems to support positive behavior change and satisfy multiple stakeholders.

## Introduction

Children are most vulnerable and at the highest risk of dying during the first 28 days of life with an estimated 2.5 million annual neonatal deaths globally [1,2]. Despite the overall reduction in the number of under-five deaths since the release of the Sustainable Development Goals (SDGs), the proportion of neonatal deaths among all under-five deaths has remained high, accounting for 47% of under-five deaths in 2018. The leading causes of neonatal mortality are preterm birth, intrapartum-related events, and neonatal infections [1].

Several policies and low-cost interventions have been implemented to reduce under-five, infant, and neonatal mortality since the introduction of the Millennium Development Goals, and subsequent SDGs. According to the 2018 Demographic and Health Survey, Zambia’s neonatal mortality rate was 27 deaths per 1,000 live births, accounting for 64% of overall infant mortality [3]. Recent studies have described the leading causes for stillbirth, neonatal, and early childhood deaths in rural Zambia and attribute almost 90% of deaths in the neonatal period to infections, prematurity, and birth asphyxia [4]. Accelerated progress is required to reduce the neonatal mortality rate by greater than 50% to meet the SDG target by 2030 [5]. A three-year roadmap (2013 to 2016) for accelerating the reduction of maternal, newborn, and child mortality was previously developed by the Zambian Ministry of Health in conjunction with other Zambian Ministries, technical workgroups, international and local non-governmental organizations, faith-based organizations, and professional societies [6]. Achieving the 2030 SDG targets as well as aligning with prior roadmaps delineated by the Zambian Ministry of Health rests on the recognition that communities and community-based structures play a critical role in providing care to families, especially those with limited access to health facilities. A deep understanding of community-based structures is required for effective and authentic partnership with communities to improve maternal, neonatal and child survival practices.

Neonatal health is linked with newborn care practices and distinct cultural perceptions of health and illness [7]. Some information is available from qualitative studies on how these cultural beliefs and experience with the healthcare system inform care-seeking behaviors, especially for newborn care practices at the community level [8–11]. Varying by Province, 33–50% of births occur in a health facility in Zambia, highlighting the importance of community perceptions regarding postnatal newborn care [3,12,13]. With the COVID-pandemic, a trend towards more home births has been noted globally and specifically across all provinces in Zambia by the Ministry of Health [14]. Past qualitative research conducted in rural Zambia described how cultural beliefs in aspects of newborn care may be either at odds with—or in some cases aligned with—clinical recommendations for newborns [12,15]. Strategies to improve neonatal survival and reduce neonatal mortality require examining how community perceptions inform care-seeking practices in order to successfully implement outcome-based, culturally accepted health interventions.

Using qualitative data collected between February and April 2010 as part of the Zambia Chlorhexidine Application Trial, Herlihy et al. investigated practices, beliefs, attitudes, and perceptions of umbilical cord care and illness in newborns [10]. Here, we aim to broaden the analysis, beyond cord care, to understand how cultural beliefs influence newborn care behaviors, perception of illness, and care-seeking practices in Zambia’s Southern Province. We aim to contribute to the growing body of knowledge on community perceptions of newborn health, illness, and care behaviors in order to improve programmatic efforts to reduce neonatal mortality in Southern Province, Zambia, and similar settings.

## Methods

The Zambia Chlorhexidine Application Trial (ZamCAT) qualitative study was conducted in Southern Province in 2010 to inform the design and procedures for a cluster-randomized controlled trial that compared daily cord cleansing with 4% chlorhexidine with the Zambian Ministry of Health recommended dry cord care practice [16,17]. The aim of the qualitative study was to identify context-specific information to aid the design and implementation of the trial, particularly around newborn care messaging, household visitation in the neonatal period, and acceptable characteristics of data collectors to the community for home visitation. A detailed study protocol, main study outcomes, perceptions of newborn care messaging, and of cord health and illness were previously published [10,16–18].

**Fig 1:**
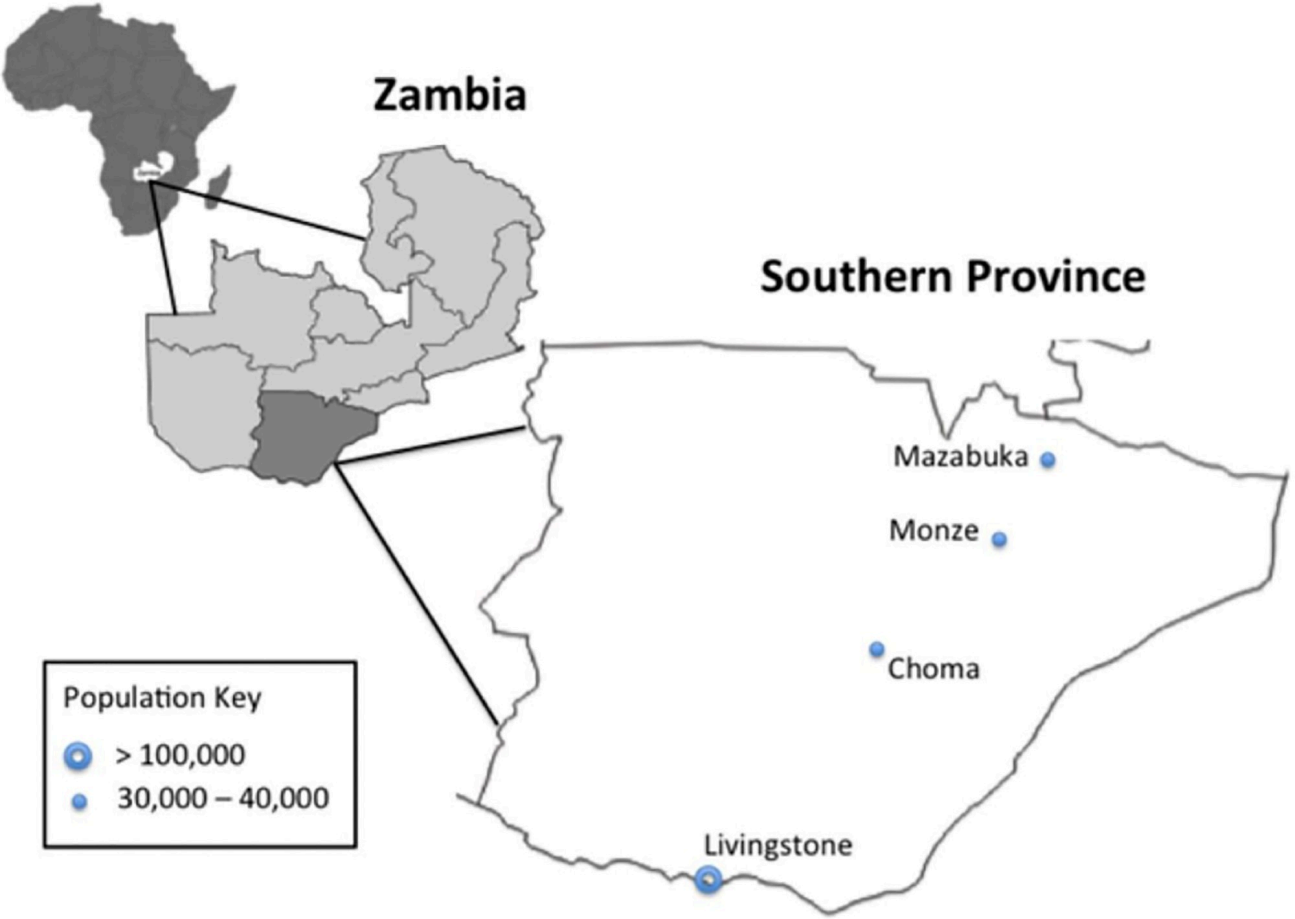
Map of Zambia’s Southern Province highlighting ZamCAT study districts [10].

Focus group discussions (FGDs) and in-depth interviews (IDIs) were conducted to determine knowledge, attitudes, and practices of mothers, traditional birth attendants (TBAs), community members, and facility-based health workers from February to April 2010. Using a semi-structured interview guide, topics discussed included general neonatal care, post-delivery practices, and neonatal umbilical cord care practices. Interviewers met with health facility staff, health committee members, and village leaders to recruit participants from four out of eleven Southern Province districts, including two rural districts (Choma and Monze) and two urban districts (Livingstone and Mazabuka) (Fig 1). FGD inclusion criteria included: breastfeeding mothers (to ensure recent birth) or grandmothers of a child under 5 years of age or traditional birth attendants (TBAs). Breastfeeding mothers and grandmothers were recruited from health facilities in the four districts. TBAs could either be graduates from the recent national training program or informal/untrained TBAs or women who are known to attend home deliveries in their communities. TBAs were identified by local headmen and a snowball method was used to identify other TBAs. Interview settings were chosen away from health facilities and without the presence of health facility or community leaders to encourage openness and reduce reporting or desirability bias. Additional criteria for study site selection and an ethnographic analysis of umbilical cord care can be found in Herlihy et al [10].

Interview guides were piloted with volunteer mothers and grandmothers. Interviewers were recruited based on background experience and were former midwives who previously worked for the government health sector and underwent training in research ethics, qualitative methods, and interviewing techniques. Interview guides were adapted post-piloting and submitted to the respective institutional review boards and ethics committees prior to use. IDIs were conducted with key informants in the community, including community and religious leaders, community health workers (CHWs), neighborhood health committee (NHC) members, health professionals, midwives, traditional healers, and trained and untrained TBAs. Most FGDs were conducted in Tonga, the predominant language in Southern Province. Two FGDs were conducted in another local dialect, Nyanja, in Livingstone District; one FGD was conducted in English with facility-based midwives.

All FGDs and IDIs were audio-recorded, translated (where needed) into English, and uploaded into NVivo software version 9.0 [19]. Four independent reviewers each coded half of the transcripts allowing for double coding of each transcript. Key themes were identified and discrepancies were reconciled during group review. Key themes focused on models of care seeking, belief constructs, and practices; emerging themes were also identified using thematic analysis [20].

### Ethics statement

The protocol and consent forms were reviewed and approved by the Boston University Institutional Review Board (FWA# 00008404) and the University of Zambia Research Ethics Committee (FWA# 00001131). The Zambian Ministry of Health also reviewed and approved the study. FGD inclusion criteria included: breastfeeding mothers (to ensure recent birth) or grandmothers of a child under 5 years of age or traditional birth attendants (TBAs). Written informed consent was obtained from each participant. Interviewers read a translated Tonga version of the consent form to all participants, in a group for FGDs or individually for IDIs. An English consent form was also available, if preferred. Participants were given an opportunity to ask clarifying questions or opt-out and conclude their participation. Consenting participants provided documentation of consent with a signature, mark, or thumbprint; copies of consent forms were provided to participants.

### Conceptual framework

**Fig 2:**
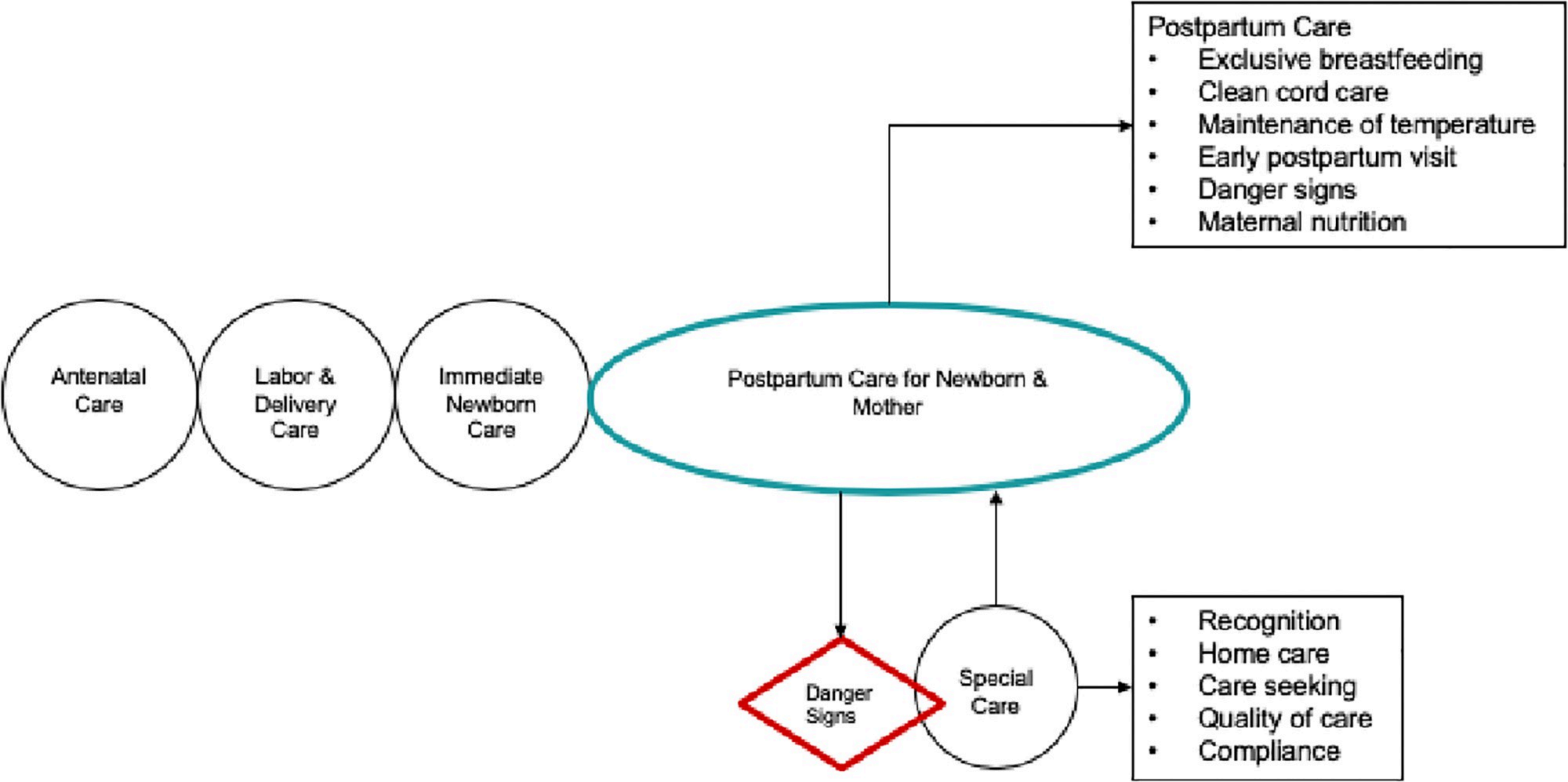
Essential elements, including emphasis on the importance of recognition of and appropriate response to illness, including timely care-seeking, in the pathway to newborn health and survival.

The conceptual framework (Fig 2) was adapted from Marsh et al., which defined positive outcomes and behaviors for “exemplary newborn care [21].” Despite the numerous logistical and financial barriers that can hinder care-seeking behaviors and access to quality care, the framework describes a pathway that, if implemented well, would result in positive outcomes across four main periods from conception, pregnancy, delivery, and postpartum care. Key behaviors emphasized for newborn health during the postpartum period include optimal nutrition, exclusive breastfeeding, hygiene, clean cord care, thermoregulation, and awareness of other newborn danger signs to ensure health and survival. If circumstances deviate from the optimal health pathway, caregivers must be able to quickly identify danger signs and symptoms of illness. Early recognition of these danger signs is crucial for immediate care-seeking, identified as “the special care” cluster, to return to the optimal care pathway. A key premise of this framework is that one must understand current practices for postpartum newborn care to ensure successful uptake and integration of evidence-based behaviors. Understanding both community norms and individual attitudes and beliefs can contextualize differences in practices and behavior-change modifications for improved newborn care.

## Results

### Participant demographics

A total of 339 women participated in 36 FGDs, with 9 FGDs conducted in each of the four study districts [Tables 1 and 2]. Among the 36 FGDs, 13 were conducted with breastfeeding mothers, 12 with grandmothers who had grandchildren under-5 years of age, 10 with trained and untrained TBAs, and 1 FGD with health facility-based midwives. Of the 42 IDIs conducted, 7 traditional birth attendants, 8 traditional healers, 15 health professionals including midwives and community health workers, and 4 religious leaders were interviewed. The IDIs were distributed across the 4 study districts; 11 IDIs each were conducted in Choma and Monze and 10 were conducted in both Livingstone and Mazabuka.

**Table 1:** Focus group discussion (FGD) participant demographic characteristics.

Focus Group Discussion Type	Number of FGDs (n = 36) n (%)	Number of participants (n = 339) n (%)	Number of Rural Participants n (%)	Median Age (years)
Breastfeeding mothers	13 (36.1)	118 (34.8)	63 (53.4)	23.5^∍^
Grandmothers	12 (33.3)	117 (34.5)	58 (49.6)	56.5^α^
Midwives	1 (2.8)	8 (2.4)	0 (0)	N/A
Trained and untrained TBAs	10 (27.8)	96 (28.3)	50 (52.1)	47.0^β^

^∍^Age data were only available for 80 mothers;

^α^Age data were only available for 64 grandmothers;

^β^Age data were only available for 77 untrained and trained TBAs.

**Table 2:** In-depth interview (IDI) participant demographic characteristics.

In-Depth Interviews	Number of IDIs (n = 42)	Median age, years (range)	% Rural	% Female
Trained TBAs	2	57.5 (57–58)	50	100
Untrained TBAs	5	49.6 (39–58)	60	100
Traditional healers	8	54.8 (47–68)	50	50
Health professionals	7	53.4 (49–61)	28.5	42.8
Community leaders	8	49.8 (42–53)	62.5	25
Community health workers (CHWs)	2	35*	100	50
Midwives	6	42.8 (33–59)	50	83.3
Religious leaders	4	51.5 (40–65)	25	0

* Age for one CHW was not available, therefore range not included.

The median age for the IDI respondents (which mainly focused on community members and leaders) was 51 years with participants ranging from 33 to 68 years of age [Table 2]. Complete age data were only available for 24 of the 36 FGD participants; median age was calculated using available data [Table 1]. Participants represented eleven different tribes with 62% of participants (n = 26) belonging to the Tonga tribe. The remaining 38% of participants were from the Ila, Kalunda, Kaonde, Lala, Lozi, Mambwe, Ngoni, Nkoya, and Nyika tribes. Female participants had an average of five children (range: 1–10 children) and male participants had seven children on average (range: 3–13 children).

### Perceptions of illness

Respondents shared varying perceptions about the role of illness and practices to prevent poor outcomes in newborns.

#### Luhumwe: Causes, prevention & treatment

A common illness mentioned during FGDs and IDIs, *luhumwe*, was defined as abdominal distention with protuberant veins in newborns. Family members believed that *luhumwe* causes continuous crying in the newborn and occurs when a pregnant or menstruating woman passes near a newborn baby. Respondents discussed a series of methods to prevent *luhumwe*. Respondents believed that “there [s]hould be some fire burning from grass on the doorway and any visitor should step on the fire [before going to] see the baby” when visitors come to see the baby” [IDI with community health member, Monze]. Some women believed that menstruating and pregnant women should not visit the baby because they may cause the baby to have symptoms such as constipation, uncontrollable crying, and a distended abdomen with evident black veins (*luhumwe)*. If menstruating or pregnant women do visit the newborn, respondents believed they should not touch the baby because they will give the baby chest pains. The family will place a spear at the entrance, hang thread from a *chitenge* (traditional cloth), or hang fiber from a *musekese* tree to alert community members to the presence of the newborn baby in the house.

Mothers and grandmothers shared many additional strategies to prevent *luhumwe* when a pregnant or menstruating woman came to visit the child including soaking local plants or soil matter in water to make a tea or broth. Examples of types of tea/broth made included: *chombolwa* (dirt from red ant hill), *silukukwe* or *roma* soil, *mavwa* leaves, *Jalamatanga* tree, marijuana seeds or *kapinga* grass. Menstruating and pregnant women would then be asked to give sips of these mixtures to the child to prevent *luhumwe*. Mothers recommended that some pregnant women should be asked to *kumolola mwana*—stretch, pull and massage—the baby’s body to help the bowels open and treat *luhumwe*. A few respondents shared an opposing belief that pregnant, menstruating, and/or unfaithful women do not have any ill effect on a newborn baby if they choose to visit or pass in proximity to the newborn.

#### Uncontrolled and continuous crying in the newborn

Caregivers had specific perceptions regarding the causes of continual or uncontrollable crying in newborns. In most cases, mothers commented that “if the baby is crying too much, then they would consult the grandmother or TBA before going anywhere” [IDI with MoH appointee, Mazabuka]. Respondents stated that they would take the baby to the health facility in situations when the baby was crying if he/she had a sunken fontanelle or exhibited symptoms of malaria. In these cases, mothers and grandmothers would first take the baby to the health facility, and if they found no improvement, then they would take the baby to the traditional healer for treatment or to a prophet for holy waters and prayers. Some respondents believed that uncontrollable crying was context dependent. Two common reasons for uncontrollable crying were that the baby needed to be given the name of a certain deceased relative or the baby was being tormented by spirits. In both examples, families believed that hospitals could not properly address these issues and would seek assistance from traditional sources of medicine. Some mothers commented that they would immediately seek care from the traditional healer if the baby was crying uncontrollably as they believed that this was a result of parents being unfaithful, someone bewitching the baby, or *hyelo* (ghosts) roaming around the baby.

#### Care-Seeking decisions

In most cases, mothers and fathers were the key players responsible for care-seeking decisions related to their newborn’s health and welfare. Parents made decisions about when and where to seek care based on their interpretation of the newborn’s condition. In some cases, grandmothers or other elderly women in the community were consulted to make decisions regarding care-seeking practices. When asked if there were certain barriers that would impact one’s ability to seek medical care, respondents commented that distance, lack of transport, and lack of money were the most common factors. Other FGD participants believed that no barriers existed, despite being prompted to whether “money, distance, maybe relatives or anything” [FGD interviewer, Livingstone] could act as barriers. Some grandmothers commented that mothers would not take their babies for medical care because of “laziness” [FGDs with mothers, untrained TBAs and grandmothers, Livingstone and Monze] and another grandmother commented, “these days, the young mothers don’t have respect for the elders [and] that is why babies die. The young mothers will not take advice from elders because of these new churches that have come up [and] they think that problems can be solved by the church” [FGD with untrained TBAs, Livingstone]. Despite whether caregivers believed that barriers existed, many healthcare workers stated that it is more common for families to seek care from traditional healers. Justifications for going to the traditional healer included “because they believe so much in traditional medicines” [FGD with untrained TBAs, Mazabuka] and “people have taken it as a habit to go to traditional healers for any problems in their home” [IDI with MoH appointee, Choma].

### Mixed perceptions of the role of traditional medicine in neonatal illness

Participants shared very mixed perceptions regarding the role of traditional medicine in preventing or addressing illnesses during the postnatal period. Some respondents believed that “some diseases [are] not healed by clinics and children will [seek care with or] show this disease is just for traditional healers.” Mothers believed that when babies exhibit certain symptoms such as *chamutwe* (bulging fontanelle) or *kayengo* (line-like mark associated with fever), these babies must be taken to a traditional healer since these conditions can only be healed by traditional healers, and not by clinicians. Family members believed that the baby would continue to exhibit symptoms, and these could cause future problems, such as not being able to talk, unless the baby was taken to someone who is able to treat the condition; this person was likely to be a traditional care provider. Participants shared different perceptions regarding when to visit traditional healers or use traditional medicine to prevent illness. Some mothers believed that “traditional medicines are not good because they bring demon spirits to the babies and can be badly affected compared to babies that don’t use traditional medicines” [FGD with mothers, Livingstone]. However, a conflicting opinion was expressed that showed preferred for care seeking to the formal health system, for example, one mother mentioned they would “go to the TBA to ask to take the baby to the clinic, especially if the baby was crying a lot” [FGD with mothers, Nakambala].

Some traditional healers shared perceptions that people sought traditional medicine after they had been to the clinic and did not see any change in their infant’s condition despite clinical medicine interventions. An untrained TBA shared that if a child is continuously crying, then the baby should be taken to the traditional healer for prevention because “maybe someone wants to witch the baby.” One respondent expressed the belief that only a traditional healer would be able to “identify a hole in the baby’s mouth and prevent someone from ever witching the child” [FGD with untrained TBA, Choma]. In contrast, health professionals believed that “in the old days, people used [traditional medicine] before and after birth, but in these modern days, nothing is being practiced” [FGD with untrained and trained TBAs, Mazabuka]. Interviews with health workers confirmed that perceptions about traditional healing were also influenced by whether families have the financial means to seek medical care at the health facility.

### Perceptions of public health sector services and newborn care-seeking

Most respondents indicated that it was common for the newborn to attend a clinic one to two weeks after birth, especially for home-based deliveries, for routine newborn examinations and vaccination (i.e. BCG). As one participant commented, many caregivers believed in the importance of taking the newborn for vaccination during the postnatal period because “if something is a prevention—like vaccination and umbilical cord care—you do it straight away” [IDI with community health worker, Monze]. All mothers agreed that they would seek care if [their] “baby cries the whole night or has abnormal diarrhea” [FGD with mothers, Nakambala]. Caregivers unanimously agreed that they would seek care from the health facility when symptoms or the newborn’s condition were obvious [and associated with certain conditions], such as when “there is a fever present, then you know that the baby might have malaria as well” [FGD with grandmothers, Monze; IDI with untrained TBA, Livingstone]. Others reported symptoms that would prompt a visit to the health facility included body hotness, abdominal pain, pus discharge or swelling of the cord, and rash.

There were, however, many instances where respondents commented that it would not be appropriate to seek immediate care at a health facility. Some participants expressed fear that there is a bad omen or spirit associated with seeking care at the clinic and believe that “when you go [to] the hospital, you will have the baby dead. Some people will have their deliveries at home [because] mothers will literally tell their daughters don’t go to the hospital for anything while you are pregnant because you might lose this pregnancy” [IDI with religious leader, Mazabuka]. Respondents perceive that there are certain diseases—like convulsions and *chamutwe*–where “you know that there will be no assistance at the clinic…so the baby should be taken to somebody (traditional healer) who knows medicine for the disease” [IDI with untrained TBA, Livingstone]. Even before seeking care at the clinic, mothers responded that “first you get into your house and pray for your baby and then take the baby to the clinic” because sometimes the baby will be healed by the time you take the baby to the clinic [FGD with mothers, Livingstone].

### Preventing perceived witchcraft in the newborn

In the Tonga tradition, burying the placenta and cord symbolizes that every person is buried twice “once on the day of birth and once on the day of death” [IDI with community health worker, Monze]. Any improper burial of both the placenta and umbilical cord was strongly tied to a suspicion of witchcraft in the newborn among those with knowledge of the burial practices. Mothers commented that “in the old days, they used to believe that the placenta was a human being” and were advised to go to the end of the house at night and bury the placenta and cord in a *bwina* (deep hole in a mound of dirt). The placenta needed to be buried carefully in the ground and respondents believed that “if you bury [the placenta] upside down (cord-side down), it is not good because it will mean that the woman will never have babies again because you have turned her fertility upside-down” [IDI with untrained TBAs, Livingstone]. Respondents shared a common belief that if the cord or placenta were carelessly thrown away, somebody would pick it up and use it for witchcraft. Another option described for the safe disposal of the placenta and cord would be to place them in a *mpako* (hole of a tree trunk) to ensure that no one finds them. Mothers believed that witchcraft would cause the mother to become barren or die or would cause the newborn baby to be born abnormally. Respondents also believed that “if a child is protected and is mixed [visited or interacts with another newborn] with one who is not protected, it can cause problems for that baby” [IDI with community leader, Mazabuka] so they would smear a mixture of *kalembula* (pounded sweet potato leaves) or tie a string around the neck or the waist to protect the baby against witchcraft and evil spirits. Participants noted that one or many of these practices were used to protect themselves and their infants.

## Discussion

Perceptions of illness and care-seeking behaviors impacted where and from whom families sought care in Southern Province, Zambia for illness prevention and management during the postnatal period. Perception of disease according to the Tonga-speaking people can be traced back to the association of traditional African and Western worlds and perceptions during the colonization period. The Tonga classify diseases of “black people” (bwa jintu) and the diseases of “white people” (bwa jinga). The diseases of the white people are also said to come from God (Leza), or said to be “natural diseases”. Their origins are unknown and do not have any moral aspect, they can be explained by western medicine [22]. While modern health systems can treat perceived “natural diseases”, it is believed that only traditional medicine can treat causes of “black man’s disease” that are usually caused by misbehavior among people [22,23]. As demonstrated in the qualitative research here, participants noted that many of the diseases could emerge from both types of causation. This belief of disease causation provides a fitting explanation to how and why parents seek care for their newborns. Sick newborns are known to deteriorate clinically quickly and suddenly which make any delay in care a priority to identify. While care-seeking for perceived newborn illness with traditional healers may not always lead to a delay in care, it would be important to partner with traditional healers to identify systems to triage and prioritize newborns at risk of acute decompensation. As noted by the study participants, perceptions of illness and cause of illness (e.g. spirits of dead ancestors) and care providers’ ability to identify and treat illnesses during the neonatal period were strongly correlated.

Despite widespread implementation and use of cost-effective intervention packages and decreased under-five mortality, neonatal mortality remains high in many resource-limited settings [24]. While this cannot be pinpointed to one sole cause, several effective interventions have failed after being implemented without recognizing the importance of illness perception, care-seeking behaviors, and the need for behavior change interventions [25–28]. In some cases, “preconceptions, attitudes, choices (or lack thereof) and traditional *know-hows*” were largely disregarded in the implementation of critical interventions to reduce the burden of neonatal mortality [29,30]. One notable example can be found in a multi-site community-based trial in Benin on scheduled screening and treatment for malaria in pregnancy. Despite having standard operating procedures, community sensitization meetings, and informed consent procedures, various perceptions regarding the role of the placenta in the community led to fears and rumors about the placenta biopsy sampling component of the study. Eventually, “the confluence of socio-political power relations, economic inequality, socio-cultural dynamics, informed consent procedures, and a catalyzing adverse event” led to the decline of the trial in Benin site [31]. Local perceptions are strongly connected to a given community’s norms and values, which may have significant impacts on health system utilization [32].

Some data are available from sub-Saharan Africa on the connection of cultural beliefs and care-seeking behaviors. Evidence exists in the literature indicating that people do not seek care, from traditional or biomedical systems, if those treatments do not meet their needs [33–35]. A mixed-methods study conducted in rural Ghana found that care-seeking is a complex process strongly influenced by health beliefs and identified several barriers to clinically appropriate care-seeking practices. Among barriers identified were a class of illnesses defined as “not-for-hospital”, which were tied to a strong belief in the efficacy of traditional medicine and culturally prescribed actions for locally named illnesses. The study concluded that interventions focusing on symptom recognition would not improve poor care-seeking behavior, since results regarding cultural beliefs and barriers (as well as teaching danger signs) can be best addressed using local terminology [36]. Other qualitative studies commented on family members’ perceptions of newborn care-seeking practices. Results from a multi-site sub-Saharan African qualitative study identified “the need to broaden interventions to engage the wider family in a meaningful way…especially first-time mothers who may be open to new practices” [37]. A qualitative postnatal care acceptability study from rural Uganda found that while most recommended newborn care practices (i.e. exclusive breastfeeding, delayed bathing, danger sign recognition, etc.) were said to be acceptable by both health providers and community members, postnatal care-seeking was poor and not promoted by service providers or through community outreach activities [38]. Improving post-natal care practices thus requires both uptake by community members as well as promotion and outreach by care providers and provision of high quality, respectful care. Approaches to partnership with community leaders and traditional healers ought to be explored in order to integrate the co-existing systems to benefit families and newborn outcomes.

More data are available from South Asia-specific studies on the influence of cultural beliefs on perceptions of newborn illness and related health care practices. Community-based trials conducted in South Asia suggest that newborn care practices can be improved through appropriate behavior change interventions. A qualitative study exploring illness care-seeking for young infants in urban India found that mothers were able to recognize infant illness (diarrhea, fever, cough, labored breathing, and muscle wasting) in a timely fashion. However, the limiting factor was mothers’ difficulty distinguishing between formally and informally trained health care sources; ultimately, they gave preference to local informally trained providers [39]. It should again be noted that families are not solely accountable for these shortcomings as health systems play an important role in promoting recommended care and ensuring positive experiences of care for families. Multiple qualitative studies conducted in Bangladesh reference the need to highlight community awareness on neonatal morbidity and the importance of care-seeking from trained personnel [40,41]. Two learnings from a qualitative study conducted in Sylhet recommended that programs should aim to provide care at home and prioritize making facility-based care more culturally acceptable in neonatal health interventions [40,41]. An implementation research study, conducted in rural Bangladesh to assess caregiver acceptability of World Health Organization (WHO) guidelines for managing possible serious bacterial infections, identified low acceptability among caregivers for referral due to distrust in hospital doctors, inconsistent medicine availability and financial constraints, unless they believed the infants’ illness to be severe. In this case, some providers developed local solutions—such as engaging village doctors in treatment to address organizational barriers and promote treatment adherence [42]. These recommendations are especially relevant to policy makers, with the Ministry of Health or at the sub-national level, since engaging with community leaders to develop culturally appropriate strategies can improve uptake and adherence to behaviors and care-seeking practices to ensure newborn health.

Though the South Asian study results may not be generalizable or deliverable to sub-Saharan African countries, they do reflect the significant influence of cultural beliefs on perceptions of newborn care and the need to address and incorporate cultural beliefs in neonatal care improvement strategies [43]. As noted in the conceptual framework by Marsh et al., community-based interventions are needed to educate women in essential newborn care and birth preparedness, illness recognition, and timely, appropriate care-seeking. Consequently, community mobilization initiatives can empower caregivers and communities to develop strategies to access care that are appropriate within the local context [21]. Home-based neonatal care is an acceptable and feasible intervention to enhance access to skilled healthcare services if barriers exist to seeking facility-based care [29]. It is important to recognize that not all healthcare problems can be addressed at home alone; access to high quality health care for maternal and neonatal care is critical. Health facility staff can engage with community-level health workers, such as CHWs and TBAs, to ensure that parents of newborns have equitable access to healthcare services by facilitating and referring families interested in seeking facility-based care when needed.

Unsurprisingly, these findings are consistent with an analysis using the ZamCAT qualitative dataset by Herlihy et al. that assessed local perceptions of umbilical cord health and illness and the cultural belief system that shapes cord care knowledge, attitudes, and practices from the same ZamCAT qualitative study population [10]. Herlihy et al. highlighted the benefit of integrating cultural practices with biomedical care in Southern Province, Zambia and noted that this blending can create opportunities for a health systems model where policy makers and program designers can find synergistic overlaps between beliefs and health systems that would both result in positive behavior change and satisfy multiple stakeholders [10]. This qualitative study’s results offered extensive guidance in the design of the intervention trial and changes to the community engagement strategy [16].

Findings presented in this analysis may be useful in further understanding common terminology (such as *chamutwe*, *kayengo*, and *luhumwe*) and beliefs around newborn illnesses from the community in Southern Province, Zambia. These findings may be applicable to other Zambian communities as evident by similarities found with the qualitative study conducted in the rural Lufwanyama District of Zambia’s Copperbelt Province assessing community perceptions and management of newborn hypothermia. Capturing local terminology, understanding patterns of care-seeking, and describing perceived sources of disease could improve future intervention studies and care-seeking pathways to optimize research and health system design.

Since the completion of the ZamCAT study in 2013, Zambia has scaled up efforts to meet SDG goals to reduce neonatal mortality. One notable endeavor included expanding an integrated community case management (iCCM) program strategy with screening and referral for sick newborns [44,45]. However, coverage and use of these interventions remains low, especially in rural communities where the interventions are most needed. Before 2018, 81–82% of deliveries took place in a health facility or were attended by a skilled health provider, but only 65% of these deliveries had a postnatal care visit within two days of delivery, which is the recommendation from Zambian Ministry of Health [3,46]. The uptake of facility-based birth services decreased further during the COVID-19 pandemic. To address this misalignment of guideline and coverage, the Zambian Ministry of Health launched the Essential Newborn Care Training Package and Newborn Protocols to assist local stakeholders using a multipronged approach to reducing neonatal mortality through training, capacity building, and provision of medical supplies in various districts [45]. This effort was later reflected through WHO policy recommendations to provide early neonatal care training for community-based agents, such as community health workers and TBAs, to greatly increase the chances for newborn survival [45].

### Strengths & limitations

Strengths of the ZamCAT qualitative study include its large sample size, leading to a diversity of participants with multiple perspectives from family members and community members across several districts of Zambia’s Southern Province. Further, this study intentionally gathered information from a variety of community groups, stakeholders, and leaders. One potential limitation of our approach, however, is that participants shared beliefs that existed in the community but may not have been their own personal beliefs, which could present an availability bias. Second, this study was conducted in the Southern Province; thus, perception of illness, newborn care behaviors and care-seeking practices may not necessarily reflect beliefs shared by communities outside the Southern Province. Third, this study did not include husbands or other male figures in the focus group discussions to obtain perspectives of men as family members/fathers/husbands; however; we did gather perspectives from male community leaders in the in-depth interviews. Further, we included grandmothers who had grandchildren born in the previous five years; there is a potential of recall bias for this sub-group. Finally, these data were gathered in 2010 and although we used the data to influence planning for the primary study, ZamCAT, we did not develop this manuscript until a decade later. Despite this time delay, these data remain aligned with more recent studies on community perceptions of birth, newborn illness and careseeking, especially from Zambia and the sub-Saharan African region [47–50]. Since home-based childbirth increased during the COVID-19 pandemic and uptake of postnatal care is relatively low in this region, we believe these data remain highly relevant to understanding community beliefs and practices in Southern Province.

## Conclusion

This qualitative study demonstrates the connection between cultural beliefs and their important role in the perception of newborn illness, newborn care behaviors, and care-seeking practices in Zambia’s Southern Province. These results were applied to inform the design and methods for the ZamCAT intervention trial. Further, these findings can be used to strategically inform future programs and policies addressing causes of neonatal mortality in Zambia, whether being implemented by a regional, national, or global organization. These efforts will be especially relevant in Zambian communities that share similar perceptions regarding the role of cultural beliefs and newborn care-seeking to the Tonga in Southern Province. The Zambian government remains committed to achieving the SDGs by 2030, and efforts are necessary to reduce the contribution of neonatal mortality to the under-five mortality rate. Stakeholders could use this information on cultural beliefs and their corresponding influence on newborn care-seeking practices to contextualize existing best practices and improve uptake of strategies that reduce the burden of illness and preventable mortality in the newborn period.

## Supporting information

S1 File (DOCX)
