# Supplementary material for: Impact of beliefs on perception of newborn illness, caregiver behaviors, and care-seeking practices in Zambia’s Southern province
Source: PLoS One. 2023 May 25;18(5):e0282881. doi: 10.1371/journal.pone.0282881 (PMC10212139; doi:10.1371/journal.pone.0282881)
Supplement: S1 File — (DOCX) [file pone.0282881.s001.docx]

**Glossary of Terms**

| *akalaso* | Pneumonia |
| --- | --- |
| *bwina* | Deep hole in a mound of dirt |
| *chamutwe* | Bulging fontanelle; often combined with continuous diarrhea or with chibele (growth in the mouth). Can be treated by tradition healers who give the babies nsimbo (tattoo) on the front and back of the head or a mixture of bubble fish head, impwa leaves and banana leaves applied to the fontanel. |
| *chitenge* | Sarong or traditional cloth |
| *chombolwa* | Dirt from red ant hill |
| *hyelo* | Ghost |
| *kalembula* | Pounded sweet potato leaves |
| *kayengo* | Line-like mark associated with fever |
| *kumolola mwana* | Stretch, pull or massage |
| *luhumwe* | Abdominal distention with protuberant veins; causes baby to cry continuously and can bring about death. Comes from contact with, or proximity to, pregnant or menstruating women. Can be prevented to treated by placing kachechete tree branch at entrance, tie chitenge cotton to baby’s wrist, rub baby’s belly, make cross on baby’s umbilicus with ash or giving baby drink of ntuntulwa (tree), kapinga (grass) or loma (soil) mixed with water. |
| *mpako* | Hole of a tree trunk |
| *mulozi* | Sorcery |
| *tsalale* | Pollution |
